# Impact of brushing-flossing sequence on plaque removal: a single-blind, randomized controlled trial

**DOI:** 10.1186/s12903-026-07984-6

**Published:** 2026-02-25

**Authors:** Yi-Fei Ma, Yi-Hui Pan, Yi Tang, Xiang-Zhen Yan

**Affiliations:** Shanghai Engineering Research Center of Tooth Restoration and Regeneration & Tongji Research Institute of Stomatology & Department of Periodontology, Shanghai Tongji Stomatological Hospital and Dental School, Tongji University, Shanghai, 200072 P. R. China Yanchang Road 399,

**Keywords:** dental plaque, flossing, brushing, randomized controlled trial, dental hygiene

## Abstract

**Background:**

Brushing and flossing have been widely used worldwide as mechanical ways for the removal of dental plaque. The aim of this study was to investigate whether the sequence of brushing and flossing would have an impact on the efficacy of plaque removal.

**Methods:**

A total of 56 college students were randomly assigned to the floss-brush group and the brush-floss group. A baseline plaque index (PI) assessment was performed on the participants, followed by oral hygiene instruction on the modified Bass brushing technique and flossing methods. The participants in the floss-brush group were directed to floss before brushing their teeth, while the brush-floss group executed the same tasks, differing only in the order of operations. The Turesky Modified Quigley-Hein Plaque Index (TMQHPI) was utilized to assess total, buccal, lingual, and interproximal plaque, with results deemed statistically significant at *p* < 0.05.

**Results:**

Values are presented as mean ± standard deviation (mean ± SD). The floss-brush group exhibited significantly greater mean ± SD reductions in PI compared to the brush-floss group in the maxillary anterior region for both total surfaces (1.13 ± 0.87 vs. 0.71 ± 0.89; *p* = 0.011) and interproximal surfaces (1.15 ± 0.92 vs. 0.65 ± 0.86; *p* = 0.01), as well as in the mandibular anterior region for total surfaces (0.91 ± 0.76 vs. 0.64 ± 0.95; *p* = 0.026) and interproximal surfaces (0.93 ± 0.80 vs. 0.62 ± 0.96; *p* = 0.031). No statistically significant difference was identified between the two groups in other regions.

**Conclusion:**

Our results suggest a potential advantage of flossing followed by brushing in anterior regions, evidenced by greater PI reduction compared to brushing then flossing.

**Trial registration:**

(23-07-2024) on https://www.chictr.org.cn/ with the ID (ChiCTR2400087214).

**Supplementary Information:**

The online version contains supplementary material available at 10.1186/s12903-026-07984-6.

## Introduction

Periodontitis, a prevalent global disease, imposes a considerable burden on public health [1]. As the primary cause of tooth loss in adults [2], its age-standardized prevalence (severe forms) increased by 8.44% worldwide from 1990 to 2019, with higher incidence in less developed countries [3]. It is therefore crucial to enhance the management of periodontitis [4]. Crucially, gingival inflammation triggered by dental plaque serves as the key precursor to periodontitis, and its mitigation is essential for periodontitis prevention [5]. Periodontitis was found to be associated with the presence and number of untreated root carious lesions [6]. Dental plaque drives both dental caries formation and gingival inflammation through self-reinforcing microbiota-host feedback loops; however, their pathogenic pathways are fundamentally distinct: dental caries is essentially driven by microbial acid production from dietary sugars, leading to hard tissue demineralization, whereas periodontal disease results from a host inflammatory response directly triggered by the plaque, ultimately causing soft tissue destruction and bone resorption [7]. Therefore, it is important to implement timely plaque control measures to intervene in the formation and development of biofilm [8]. Mechanical plaque removal has been demonstrated to be an effective method for destroying the plaque and halting further deterioration of periodontal tissues [9].

Brushing as the primary cleaning method has been globally endorsed as an effective and straightforward approach to plaque removal [10, 11]. Nevertheless, it is challenging to effectively clean the interproximal.

surfaces of the teeth with a toothbrush alone. The interproximal surfaces of teeth are conducive to bacterial colonization and are also optimal sites for the development of periodontal disease and dental caries; therefore, the utilization of interproximal cleaning instruments is of great importance [12, 13]. Interproximal cleaning can be achieved using different devices, including interdental brushes (IDBs) and dental floss. Various IDB shapes and sizes are required in clinical practice to accommodate different interdental spaces. The improper selection or use of IDBs poses a risk of trauma [14]. In young individuals in whom the papillae fill out the interdental spaces, dental floss is the only tool that can reach into this area. Therefore, this study chose dental floss as the tool to be used for cleaning between the teeth [15, 16]. Flossing represents the optimal method for individuals with close contact points and limited interproximal space [17]. A meta-analysis of the data included by Sambunjak et al.. demonstrated that flossing combined with brushing was an effective method for reducing gingivitis compared with brushing alone [18].

The question of whether to floss before or after brushing is pertinent; however, minimal public study has been conducted to address it. Many patients wonder about the order of brushing and flossing, as these are the most often utilized mechanical methods for plaque removal. Does the sequence of brushing and flossing affect the plaque removal? We found this topic captivating. The aim of our study is to investigate whether the different sequences of brushing and flossing have an impact on plaque index (PI) reduction. The null hypothesis was that there would be no difference in plaque reduction between the floss-brush and brush-floss sequences.

## Materials and methods

### Sample selection

This study was approved by the Shanghai Tongji Stomatological Hospital and Dental School of Tongji University (Approval Number: [2024]-SR-48 dated 10/07/2024) and adhere to CONSORT guidelines for reporting clinical trials [19]. It was registered at the Chinese Clinical Trials Registry with a registration number of ChiCTR2400087214 (23/07/2024). A single-blind, randomized, parallel-controlled clinical trial study was conducted on students enrolled in the College of Stomatology, Tongji University. A confidence level of 95% and a statistical test power of 80% were considered for the study [20]. Drawing on the findings of previous literature (mean ± SD: 0.634 ± 0.07 for the floss-brush group and 0.542 ± 0.14 for the brush-floss group), the sample size was calculated based on a two-sample comparison of means using the formula for continuous outcomes:

$$\:[n=\frac{2\cdot\:({Z}_{1-\alpha\:/2}+{Z}_{1-\beta\:}{)}^{2}\cdot\:{\sigma\:}^{2}}{{\delta\:}^{2}}]$$

**Fig. 1:**
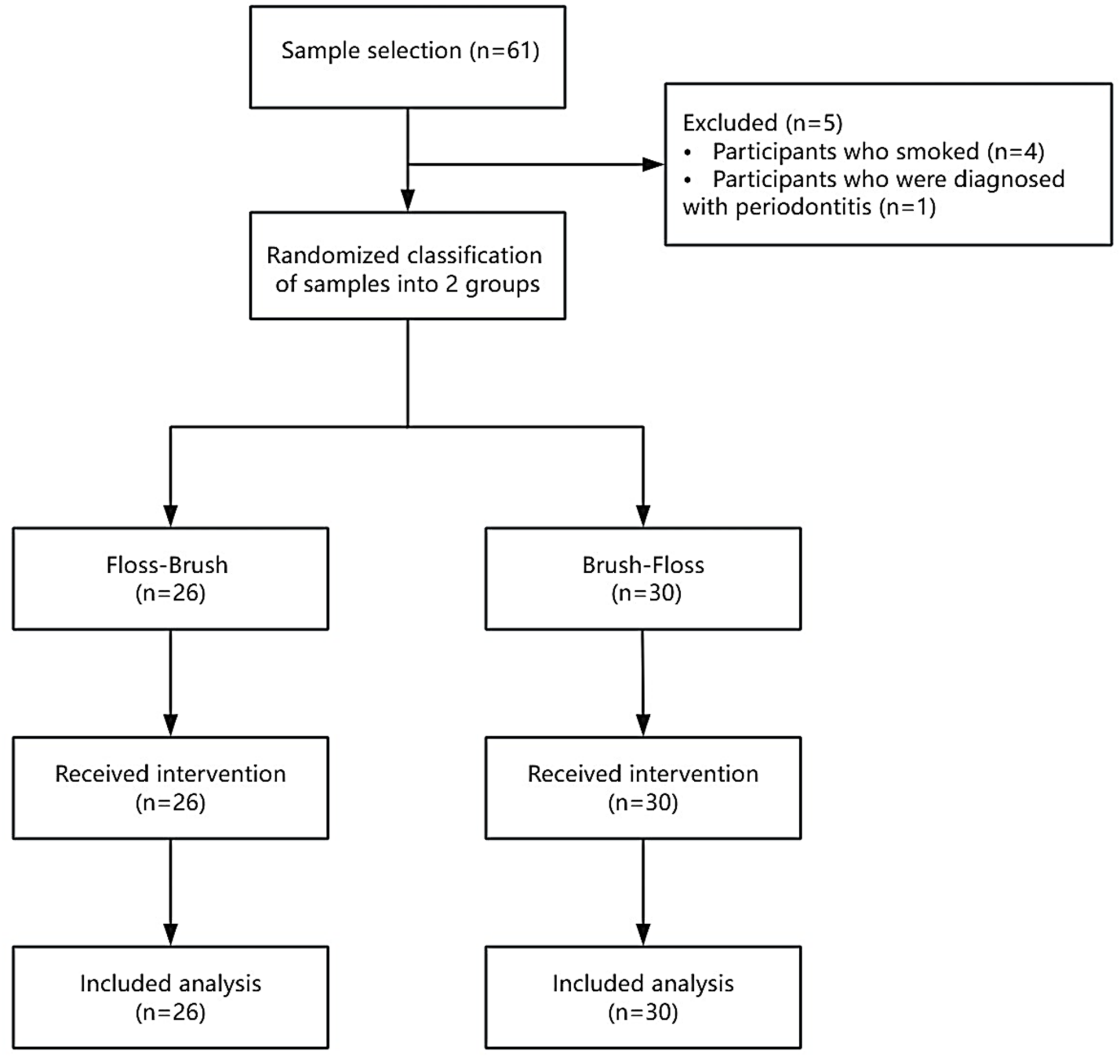
Flow chart of experimental design

24 samples for each group were calculated [21]. The inclusion criteria were as follows: (1) Individuals willing to participate and demonstrating full adherence to the study protocol, assessed via direct observation of technique execution and session attendance. (2) Healthy individuals without systemic disease, as well as salivary disorders. (3) Individuals who have at least twenty teeth, with at least five teeth and four interdental areas in each quadrant, including two in the molar region, two in the premolar region, and two in the anterior region. Individuals with missing teeth solely due to orthodontic extraction who do not have missing tooth spaces are not defined as having missing teeth. (4) Individuals who are not pregnant. The exclusion criteria were as follows: (1) Individuals who are current smokers. (2) Individuals who have periodontitis. Prior to their involvement, demographic parameters were collected for characterization and group comparability assessment but were not used as exclusion criteria. All participants were required to sign an informed written consent form. The principal investigator supervised the randomization process. Computer-based randomization was performed using Random.org’s coin flip simulator, which generates true randomness via atmospheric noise. Each virtual coin toss (Heads=Floss-Brush, Tails=Brush-Floss) determined group assignment. The participants were randomly assigned to two groups: a floss-brush group (*n* = 26) and a brush-floss group (*n* = 30) (Figure. 1). The fundamental characteristics of the participants were as follows (Table 1).

**Table 1:** Fundamental characteristics of the participants. “Healthy” is defined as < 10% bleeding sites with probing depths ≤ 3 mm. “Gingivitis” is defined as ≥ 10% bleeding sites with probing depths ≤ 3 mm [22]

	Floss-Brush	Brush-Floss	*p*-value
	*n* = 26	*n* = 30	
Gender			0.890
Male	9 (34.6%)	12 (40.0%)	
Female	17 (65.4%)	18 (60.0%)	
Periodontal status			0.576
Healthy	17 (68.0%)	22 (78.6%)	
Gingivitis	8 (32.0%)	6 (21.4%)	
Alcohol intake			0.675
No	24 (92.3%)	26 (86.7%)	
Yes	2 (7.69%)	4 (13.3%)	
Sugar-sweetened beverage consumption			0.875
No	15 (57.7%)	19 (63.3%)	
Yes	11 (42.3%)	11 (36.7%)	
Dental floss use			0.455
No	19 (73.1%)	18 (60.0%)	
Yes	7 (26.9%)	12 (40.0%)	

### Experimental protocol

Each participant was provided with a medium-bristle toothbrush (Colgate-Palmolive; New York, NY, USA), a fluoride toothpaste (Saky; Shanghai, China), and a 40-cm piece of dental floss (Oral-B; Procter & Gamble; Cincinnati, OH, USA). in the following trial (Supplementary Figure. 1). The modified Bass brushing technique and appropriate flossing instruction were taught using videos and model demonstrations. The participants were senior-year students of the College of Stomatology, which ensured that their basic understanding was similar. Preoperative and postoperative plaque index (PI) assessments were performed using a plaque-disclosing agent (CI, Japan) applied to buccal, lingual, and interproximal surfaces, followed by 30-second rinsing. Subsequently, participants in the floss-brush group cleaned their interproximal surface for a period of two minutes with the floss, rinsed their mouths routinely, and brushed their teeth for three minutes using the modified Bass brushing method. The brush-floss group performed the same procedure as the floss-brush group, except that the participants were asked to floss and rinse after brushing. Examiners recorded baseline and postoperative PI for all participants. All three examiners underwent rigorous calibration prior to study initiation. The first volunteer in every group of ten was re-examined by an examiner with > 10 years of clinical experience to evaluate inter-examiner reliability for all variables. Additionally, the total number of missing teeth in both groups was documented. Procedures were performed in standardized dental operatories. Participants used front-facing mirrors during all procedures. This study was conducted using the single-blind method, whereby only the examiners were blinded to the sequence of plaque control. It was not feasible to blind the participants.

### Plaque assessment

**Fig. 2:**
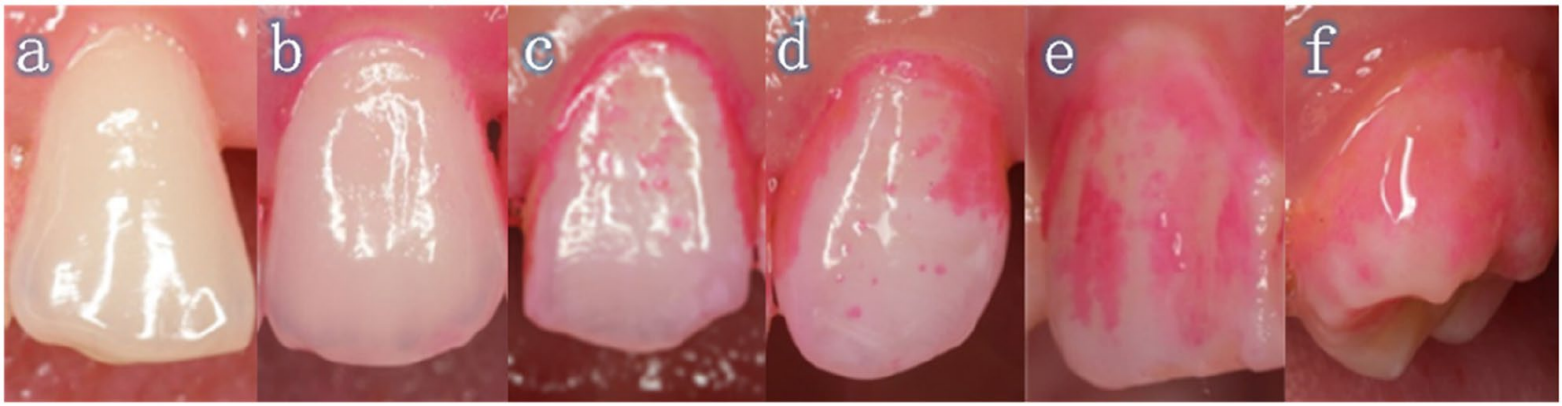
Schematic illustration of the TMQHPI. The TMQHPI scores plaque as **a** (score = 0), **b** (score = 1), **c** (score = 2), **d** (score = 3), **e** (score = 4), **f** (score = 5)

Unlike the Rustogi Modified Navy Plaque Index (RMNPI), which scores plaque presence/absence, the Turesky Modified Quigley-Hein Plaque Index (TMQHPI) quantifies plaque reduction [23]. In this study, TMQHPI was employed to assess the plaque levels of the participants prior to and after using flossing and brushing. This entailed the scoring of the distal-buccal, mesio-buccal, interproximal-buccal, distal-lingual, mesio-lingual, and interproximal-lingual surfaces of all the participants’ permanent teeth. The range of scores was recorded as follows: 0’ = No plaque present. 1’=Separate flecks of plaque at the cervical margin. 2’ = A thin continuous back of plaque (up to 1 mm) at the cervical margin. 3’ = A band of plaque wider than 1 mm but covering less than one-third of the crown. 4’ = Plaque covering at least one-third but less than two-thirds of the crown. 5’ = Plaque covering two-thirds or more of the crown (Figure 2). The mean TMQHPI scores for anterior (incisors/cuspids) and posterior (premolars/molars) regions were calculated using the formula: Total plaque scores of all examined surfaces / Total number of surfaces examined. The reduction of PI was calculated by subtracting the preoperative PI from the postoperative PI. The classification of dental surfaces was defined as follows: total, buccal, lingual, and interproximal surfaces in the maxillary anterior region, maxillary posterior region, mandibular anterior region, and mandibular posterior region, respectively.

### Statistical analysis

The statistical analyses were conducted using SPSS 27.0 (IBM; Armonk, NY, USA). Descriptive statistics are presented as mean ± standard deviation. The normality of the continuous data was assessed using the Kolmogorov-Smirnov test. As the data were found to be non-normally distributed, non-parametric tests were employed for subsequent analyses. The Levene’s test was used to assess homogeneity of variance. Categorical variables were compared by Pearson’s chi-squared test or Fisher’s exact test. Finally, the Mann-Whitney test was utilized for the comparison of the floss-brush group and the brush-floss group, as well as the full data set with the subgroup excluding individuals with missing teeth. The Wilcoxon paired signed-rank test was utilized for comparison of the reduction in PI before and after the intervention in both groups. Statistical significance level was declared at *p* < 0.05.

## Results

**Fig. 3:**
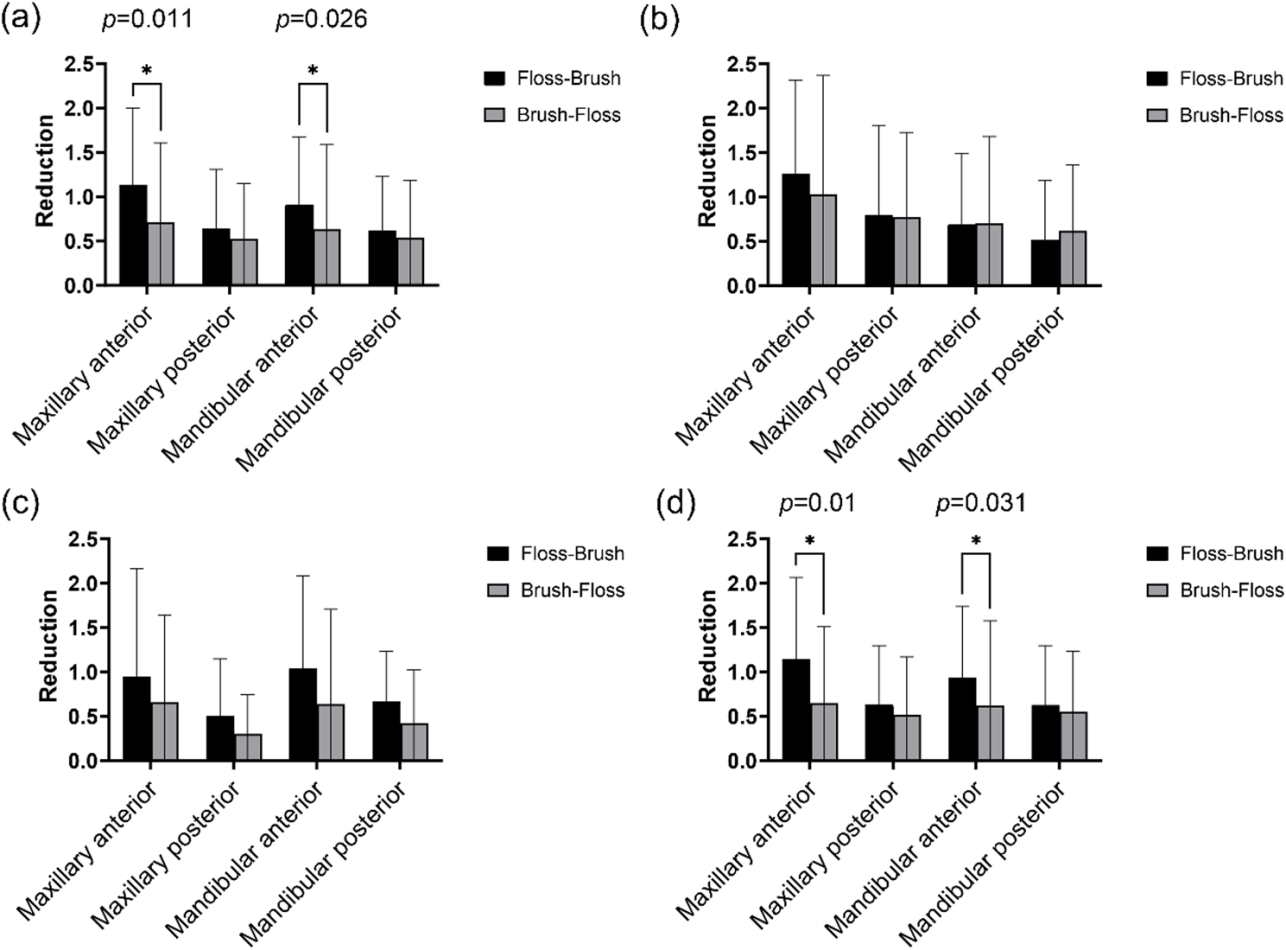
Comparison of the reduction in plaque on each tooth surface in the maxillary anterior, maxillary posterior, mandibular anterior, and mandibular posterior regions before and after plaque removal in the two groups (**a**) all surfaces; (**b**) the buccal surfaces; (**c**) the lingual surfaces (**d**)the interproximal surfaces (*p < 0.05)

A total of 56 participants were included in the study, with 42.9% identifying as female and 57.1% as male. The floss-brush group had 717 teeth, with three posterior missing teeth. The brush-floss group had 819 teeth, with seven missing teeth: six posterior and one anterior (*p* > 0.05). Table 2 presents the mean values for the pre- and post-mechanical mean PI reduction in the floss-brush and brush-floss groups. The pre- and post-operative indices for both groups demonstrated a statistically significant reduction in PI (*p* < 0.001). However, the comparison between the two groups showed a p-value of 0.055 for PI reduction. Table 3 presents the mean values of PI on buccal, lingual, and interproximal surfaces and the reduction of PI in the maxillary anterior, maxillary posterior, mandibular anterior, and mandibular posterior regions, respectively. Additionally, it illustrates the reduction in PI between the two groups. The floss-brush group showed significantly greater PI reductions than the brush-floss group in the maxillary anterior region for both total surfaces (mean ± SD: 1.13 ± 0.87 vs. 0.71 ± 0.89; *p* = 0.011) and interproximal surfaces (mean ± SD: 1.15 ± 0.92 vs. 0.65 ± 0.86; *p* = 0.01), and in the mandibular anterior region for total surfaces (mean ± SD: 0.91 ± 0.76 vs. 0.64 ± 0.95; *p* = 0.026) and interproximal surfaces (mean ± SD: 0.93 ± 0.80 vs. 0.62 ± 0.96; *p* = 0.031). The mean PI of the total tooth surfaces (*p* = 0.011 and *p* = 0.01, respectively) and interproximal surfaces (*p* = 0.026 and *p* = 0.031, respectively) in the maxillary and mandibular anterior regions in the floss-brush group were significantly greater than that of the brush-floss group. No statistically significant difference was identified between the two groups in other regions. Figure 3 illustrates the reduction of PI of each tooth surface in the maxillary anterior, maxillary posterior, mandibular anterior, and mandibular posterior regions. Given the potential impact of tooth loss on plaque removal at interproximal surfaces of adjacent teeth, Table 4 presents a comparison of interproximal PI reduction between the full analysis set and the subgroup excluding edentulous patients. The findings indicate no statistically significant impact of edentulous status on the interproximal plaque reduction process.

**Table 2:** Mean ± standard deviation (SD) of the reduction in total plaque score of the whole mouth before and after plaque removal in both groups

Total plaque results				
	Before	After	*P*-value	Reduction
Floss-Brush (mean ± SD)	1.10 ± 0.73	0.30 ± 0.32	< 0.001	0.80 ± 0.61
Brush-Floss (mean ± SD)	0.89 ± 0.82	0.29 ± 0.41	< 0.001	0.60 ± 0.71
*P-value*	> 0.05	> 0.05	-	0.055

**Table 3:** The mean ± standard deviation (SD) of reduction in plaque scores on each tooth surface was calculated for both groups before and after plaque removal in the maxillary anterior region, maxillary posterior region, mandibular anterior region, and mandibular posterior region

		Maxillary anterior teeth				Maxillary posterior teeth				Mandibular anterior teeth				Mandibular posterior teeth			
		All	Buccal	Lingual	Interproximal	All	Buccal	Lingual	Interproximal	All	Buccal	Lingual	Interproximal	All	Buccal	Lingual	Interproximal
Floss-Brush (mean ± SD)	Before	1.45 ± 0.96	1.46 ± 1.12	1.49 ± 1.38	1.50 ± 1.00	0.86 ± 0.85	1.04 ± 1.14	0.66 ± 0.86	0.87 ± 0.86	1.23 ± 0.83	0.95 ± 0.99	1.39 ± 1.25	1.26 ± 0.84	0.94 ± 0.80	0.68 ± 0.80	1.10 ± 0.97	0.96 ± 0.84
	After	0.36 ± 0.33	0.20 ± 0.36	0.54 ± 0.66	0.35 ± 0.33	0.23 ± 0.32	0.25 ± 0.40	0.15 ± 0.34	0.24 ± 0.33	0.32 ± 0.45	0.26 ± 0.57	0.35 ± 0.62	0.33 ± 0.46	0.32 ± 0.48	0.17 ± 0.34	0.44 ± 0.67	0.33 ± 0.49
	*P-value*	< 0.001	< 0.001	< 0.001	< 0.001	< 0.001	< 0.001	< 0.001	< 0.001	< 0.001	< 0.001	< 0.001	< 0.001	< 0.001	< 0.001	< 0.001	< 0.001
	Reduction	1.13 ± 0.87	1.26 ± 1.06	0.95 ± 1.22	1.15 ± 0.92	0.64 ± 0.67	0.80 ± 1.01	0.50 ± 0.64	0.63 ± 0.67	0.91 ± 0.76	0.69 ± 0.80	1.04 ± 1.04	0.93 ± 0.80	0.61 ± 0.61	0.52 ± 0.67	0.66 ± 0.57	0.63 ± 0.67
Brush-Floss (mean ± SD)	Before	1.07 ± 1.05	1.34 ± 1.42	1.06 ± 1.25	1.01 ± 1.04	0.74 ± 0.75	0.91 ± 1.08	0.52 ± 0.59	0.75 ± 0.79	0.93 ± 1.06	0.92 ± 1.02	0.91 ± 1.26	0.94 ± 1.09	0.86 ± 0.83	0.78 ± 0.82	0.72 ± 0.88	0.92 ± 0.91
	After	0.36 ± 0.47	0.31 ± 0.53	0.40 ± 0.68	0.36 ± 0.48	0.22 ± 0.39	0.14 ± 0.28	0.22 ± 0.42	0.24 ± 0.45	0.30 ± 0.42	0.22 ± 0.33	0.27 ± 0.56	0.32 ± 0.46	0.32 ± 0.54	0.16 ± 0.27	0.30 ± 0.67	0.37 ± 0.62
	*P-value*	< 0.001	< 0.001	< 0.001	< 0.001	< 0.001	< 0.001	< 0.001	< 0.001	< 0.001	< 0.001	< 0.001	< 0.001	< 0.001	< 0.001	< 0.001	< 0.001
	Reduction	0.71 ± 0.89	1.03 ± 1.34	0.66 ± 0.98	0.65 ± 0.86	0.52 ± 0.63	0.77 ± 0.96	0.30 ± 0.45	0.51 ± 0.66	0.64 ± 0.95	0.70 ± 0.98	0.64 ± 1.07	0.62 ± 0.96	0.54 ± 0.64	0.61 ± 0.74	0.42 ± 0.61	0.55 ± 0.68
*P-value*		0.011	> 0.05	> 0.05	0.01	> 0.05	> 0.05	> 0.05	> 0.05	0.026	> 0.05	> 0.05	0.031	> 0.05	> 0.05	> 0.05	> 0.05

**Table 4:** Mean ± standard deviation (SD) of the interproximal PI reduction between the full data set and the subgroup excluding edentulous individuals

Group	Region	Full analysis set	Subgroup without missing teeth	*P*-value
Floss-Brush (mean ± SD)	Maxillary anterior teeth	1.15 ± 0.92	1.15 ± 0.95	0.938
	Maxillary posterior teeth	0.63 ± 0.67	0.66 ± 0.68	0.861
	Mandibular anterior teeth	0.93 ± 0.80	0.92 ± 0.83	0.899
	Mandibular posterior teeth	0.63 ± 0.67	0.66 ± 0.68	0.800
Brush-Floss (mean ± SD)	Maxillary anterior teeth	0.65 ± 0.86	0.66 ± 0.89	0.954
	Maxillary posterior teeth	0.51 ± 0.66	0.55 ± 0.70	0.921
	Mandibular anterior teeth	0.62 ± 0.96	0.69 ± 1.01	0.651
	Mandibular posterior teeth	0.55 ± 0.68	0.60 ± 0.72	0.818

## Discussion

The European Federation of Periodontology emphasized the importance of periodontitis prevention, which is closely related to patients’ awareness of oral health [24]. The aim of this single-blind, randomized, parallel-controlled clinical trial is to ascertain whether the sequence of flossing and brushing affects plaque removal. The null hypothesis of no difference between sequences was partially rejected, as our findings suggest the floss-brush group showed significantly greater PI reductions than the brush-floss group on both total and interproximal surfaces in maxillary and mandibular anterior regions. A non-significant trend toward greater PI reduction with this sequence was observed on all surfaces. While this borderline effect may hold clinical relevance for targeted interventions, verification in larger cohorts is warranted.

The results partially aligned with findings by Torkzaban et al. and Mazhari et al. [21, 25]. They found that flossing followed by brushing provides more statistically significant improvements in PI reduction [21, 25]. However, Silva et al.‘s meta-analysis integrating these studies found no statistical difference in PI reduction between sequences, underscoring the need for further investigation [26]. The methodological heterogeneity observed between the selected studies and the limited number of studies available for quantitative synthesis might lead to this discrepancy.

Our gender-balanced cohort showed no sex-based divergence. While Torkzaban et al. incidentally observed males’ overzealous cleaning in subgroup analyses [25]. In contrast, females exhibiting superior oral hygiene practices and consistent habits demonstrated more modest plaque index reductions [25, 27]. This discrepancy may stem from our standardized hygiene protocol minimizing behavioral extremes. Mazhari *et al.‘s* RMNPI analysis demonstrated that floss-brushing sequences enhance interdental debridement efficacy through mechanical particle displacement [21]. However, their exclusively female cohort and restricted plaque assessment (interproximal/total only) limit comparative generalizability [21]. Gender constitutes a potential risk factor for oral hygiene behaviors. Females demonstrated higher interdental cleaning adherence, while male gender was identified as an independent risk factor for gingival bleeding [28, 29].

Our findings demonstrate significantly greater PI reduction in the maxillary and mandibular anterior regions with flossing before brushing. Contrary to the intuitive assumption that the plaque removal was sequence-independent, the observed anterior benefit may be explained by several factors. The anterior teeth’s anatomical position facilitates direct visual guidance during flossing, and their relatively narrower interproximal spaces more effectively facilitate the physical removal rate of dental plaque [30]. Potential differences in the composition of the dental plaque microbiota and the dynamics of salivary flow between the anterior and posterior regions may also influence cleaning efficacy [31]. The posterior region demonstrates no significant advantage, primarily attributed to its complex anatomical morphology (such as root furcations and pronounced curvature of contact surfaces), which restricts effective floss contact and demands higher technical proficiency.

The advantage of our study is that the permanent dentition is divided into four regions, which allows for a more nuanced statistical analysis of each region and a statistical comparison of the reduction in PI on each surface. Furthermore, with regard to the design of experiments, crossover studies are more time-consuming and necessitate a specific washout interval. In contrast, parallel trials may be a more cost-effective and convenient approach [32].

However, several limitations should be acknowledged. This study is limited to dental students—a population with superior oral health literacy that may amplify intervention efficacy, though generalizing findings beyond this group requires caution. Only 36.7% of participants flossed daily, and baseline hygiene habits affect manual dexterity acquisition during trial. Furthermore, participants were restricted to periodontally healthy/gingivitis individuals to eliminate pathological confounding factors; future research should therefore examine plaque control efficacy in periodontitis populations. Methodologically, plaque scoring was conducted under standard lighting, avoiding red light which is known to slow the degradation of the disclosing agent. This could potentially lead to an underestimation of plaque scores if degradation occurred more rapidly during assessment [33]. Furthermore, while the use of a plaque-disclosing agent was necessary for standardized measurement, it may have introduced a performance bias. The high visibility of stained plaque likely motivated participants to clean more thoroughly than usual, which could amplify the observed plaque removal and reduce the generalizability of the findings to routine, non-disclosed home care. Finally, while the study was adequately powered for whole-mouth plaque reduction analyses, the sample size calculation did not account for region-specific subgroup comparisons; subsequent trials should conduct a priori power analyses for key subgroups when testing site-specific hypotheses.

## Conclusion

In conclusion, this study demonstrates that flossing before brushing provides a more effective reduction of dental plaque in anterior regions than the reverse sequence. This indicates that the order of mechanical cleaning is a modifiable factor which can enhance oral hygiene efficacy, specifically for anterior teeth. Verifying this effect in broader populations and evaluating its long-term impact on clinical periodontal outcomes are necessary next steps.

## Supplementary Information

Supplementary Material 1.

Supplementary Material 2.

## Data Availability

Data is provided within the manuscript or supplementary information files.

## Abbreviations

PI: Plaque index
IDB: Interdental brush
TMQHPI: Turesky Modified Quigley-Hein Plaque Index
RMNPI: Rustogi Modified Navy Plaque Index
