# Supplementary material for: Impact of brushing-flossing sequence on plaque removal: a single-blind, randomized controlled trial
Source: BMC Oral Health. 2026 Feb 25;26:565. doi: 10.1186/s12903-026-07984-6 (PMC13040975; doi:10.1186/s12903-026-07984-6)
Supplement: Supplementary file 2 — Supplementary Material 2. [file 12903_2026_7984_MOESM2_ESM.docx]

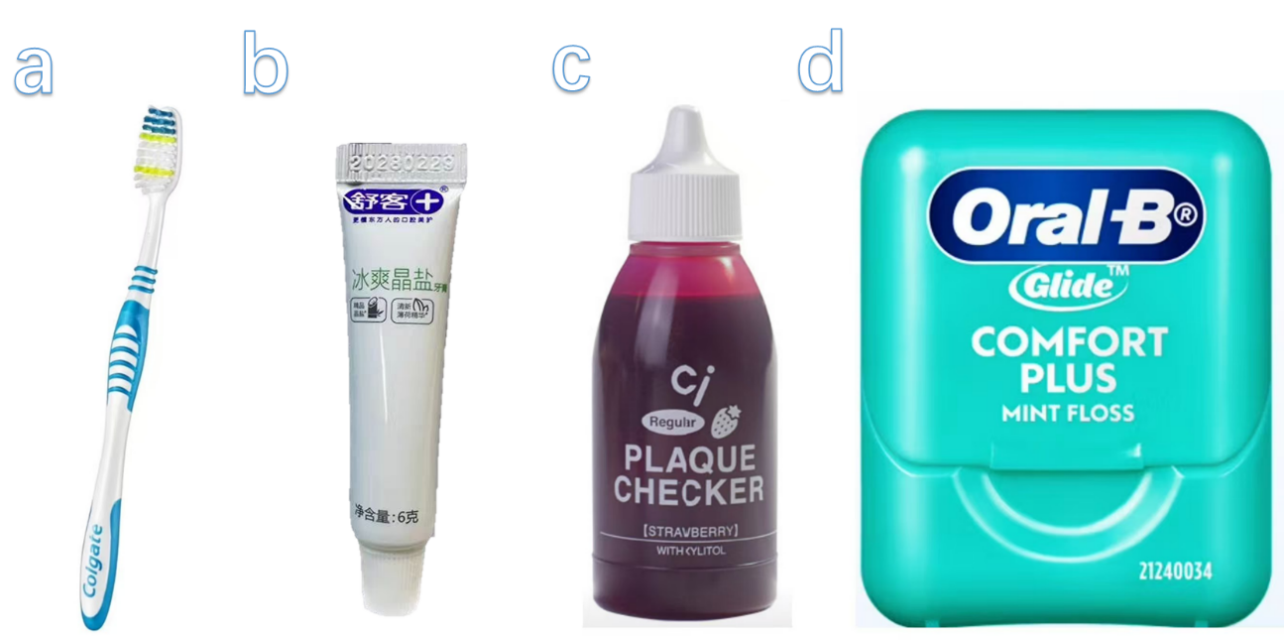


Supplementary Figure 1. Tools used in the study: a. toothpaste; b. toothbrush; c. dental floss; d. plaque indicator


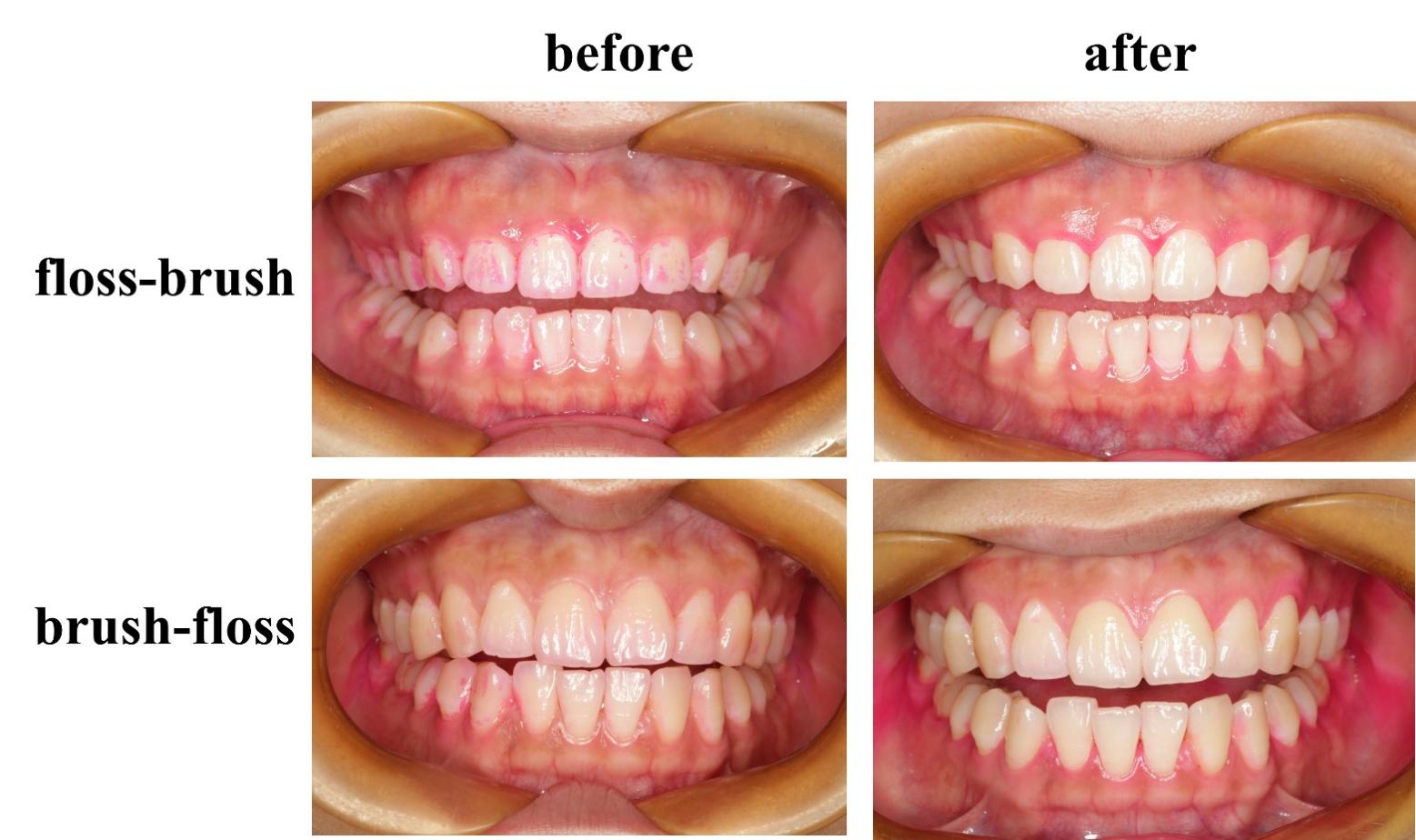


Supplementary Figure 2. Changes before and after intervention: floss-brush and brush-floss.
